# Interannual differences in pollinator contributions to pollen transfer are mainly driven by changes in pollinator abundance

**DOI:** 10.1093/aobpla/plaf009

**Published:** 2025-02-22

**Authors:** Martin Freudenfeld, Jakub Štenc, Jiří Hadrava, Michael Mikát, Eva Matoušková, Klára Daňková, Tomáš Jor, Tadeáš Ryšan, Klára Koupilová, Jan Simon-Pražák, Tomáš Dvořák, Zdeněk Janovský

**Affiliations:** Department of Botany, Faculty of Science, Charles University, Benátská 2, 128 41 Prague, Czech Republic; Department of Botany, Faculty of Science, Charles University, Benátská 2, 128 41 Prague, Czech Republic; Department of Population Biology, Institute of Botany, Czech Academy of Sciences, Lesní 322, 252 43 Průhonice, Czech Republic; Department of Zoology, Faculty of Science, Charles University, Viničná 7, 128 41 Prague, Czech Republic; Department of Zoology, Faculty of Science, Charles University, Viničná 7, 128 41 Prague, Czech Republic; Department of Zoology, Faculty of Science, Charles University, Viničná 7, 128 41 Prague, Czech Republic; Department of Zoology, Faculty of Science, Charles University, Viničná 7, 128 41 Prague, Czech Republic; Department of Zoology, Faculty of Science, Charles University, Viničná 7, 128 41 Prague, Czech Republic; Department of Zoology, Faculty of Science, Charles University, Viničná 7, 128 41 Prague, Czech Republic; Rovná 1333, Sulice, 25168, Czech Republic; Department of Zoology, Faculty of Science, Charles University, Viničná 7, 128 41 Prague, Czech Republic; Museum of Eastern Bohemia in Hradec Králové, Eliščino nábřeží 465, 500 03 Hradec Králové 3, Czech Republic; Správa Národního parku Podyjí, Na Vyhlídce 5, 669 02 Znojmo, Czech Republic; Svatý Jan t. Krsovice 1, 285 04 Uhlířské Janovice, Czech Republic

**Keywords:** pollination, pollen load, pollen transfer, pollinator abundance, conspecific pollen, pollinators

## Abstract

With the rising threat to insect pollinators and the upcoming pollinator crisis, it is important to know how pollinators contribute to pollen transfer. The contributions of individual pollinator taxa to pollen transfer depend both on their abundance and on how much pollen each individual can carry, with overall importance being a multiplication of these two values. Here, we quantified pollen load across a diverse spectrum of insect pollinator taxa and variation in their abundance over 11 years. We found that, while variation in pollen load was relatively small among pollinator taxa (compared to relatively high variability among individuals within each insect taxon), the visitation levels changed significantly over the years, resulting in a high degree of variation in pollinator contributions to pollen transfer of each insect taxon at the community level. Thus, we conclude that the overall importance of pollinator taxa for pollen transfer is determined further by their abundances than by their taxon-specific capability for carrying various pollen loads. As the insect abundances vary over time and may change dramatically from year to year, our results highlight the importance of diverse and species-rich pollinator communities, as the population decline of one pollinator can be buffered by an increase in another pollinator taxa.

## Introduction

Most flowering plants in temperate ecosystems are pollinated by insect pollinators (Ollerton *et al.* 2011) that are recruited from a wide spectrum of insect groups, which contribute to pollen transfer and plant reproductive success (Orford *et al.* 2015; Rader *et al.* 2016). With rising threats to the majority of insect pollinators (Ollerton 2017; Sánchez-Bayo and Wyckhuys 2019) and the resulting pollination crisis (Aizen *et al.* 2022), it is crucial to identify how particular pollinators contribute to pollen transfer. However, the contributions of specific pollinator taxa vary significantly due to differences in their ability to carry pollen and variations in their visitation density (Ne’eman *et al.* 2010). First, the ability of pollinators to carry pollen depends on a set of pollinator traits, e.g. body size, hairiness and feeding behaviour, which differ among pollinators (Haslett 1989; Willmer and Finlayson 2014; Phillips *et al.* 2018; Roquer-Beni *et al.* 2020; Cullen *et al.* 2021). Second, pollinator density, i.e. abundance in relation to the number of flowering plants, may vary between pollinator taxa due to differences in population size and visitation activity. Moreover, the densities of individual pollinator taxa vary significantly among years due to rapid changes in insect population sizes (Boggs 2016), causing stochastic changes in the composition of the pollinator community and turnover in plant–pollinator interactions (Herrera 2019; Thomson 2019). Consequently, differences in the ability to transfer pollen and changes in pollinator density may cause a high degree of variation in pollinator contributions to pollen transfer from year to year. Understanding how pollinator contributions to pollen transfer vary over time is crucial to our understanding of the long-term dynamics of plant–pollinator communities.

However, the comparison of pollinator contributions to pollen transfer at the community level are challenging and demanding (Minnaar *et al.* 2019). Challenges arise from the lack of techniques to track pollen fates without excessive manipulation or using expensive methods, and the number of possible plant–pollinator combinations to be dealt with in species-rich communities. Additionally, multiple approaches have been used to compare pollinator ability to transfer pollen, starting with visitation observations (Thompson 2001), comparing pollinator removal and delivery of pollen from and to flowers (Thomson and Goodell 2001; Parker *et al.* 2016) to comparing the number of seeds that developed after pollinator visits (Kandori 2002; Sahli and Conner 2007). Single observations of pollinator visitation are considered a poor proxy for pollen transfer (King *et al.* 2013). They also usually fail to indicate whether the ‘pollinator’ is not just a ‘visitor’ or even a ‘robber’ (King *et al.* 2013; Popic *et al.* 2013). Moreover, comparisons of pollen removal, deposition and seed development are time consuming and usually difficult to conduct in species-rich systems (but see Ballantyne *et al.* 2015, 2017). Another possible proxy for pollen transfer is the analysis of pollen carried on the body of pollinator, i.e. pollen load (Escaravage and Wagner 2004; Bartomeus *et al.* 2008; Alarcón 2010; Rader *et al.* 2011). Pollen load is usually obtained by gently swabbing pollinator bodies with fuchsine-stained cubes of glycerine jelly, which collect pollen grains that can then be more easily detected, identified and counted (Beattie 1971). The pollen load can be used as a rough proxy of pollen deposition on the stigma (Larsson 2005; Phillips *et al.* 2018) and can therefore be used to measure pollinator performance (Ne’eman *et al.* 2010). Moreover, pollen load can be used for comparing pollen quantity and quality in terms of the proportion of conspecific and heterospecific pollens that are transferred (Adler and Irwin 2006; Rammell *et al.* 2019).

The second important variable for estimating pollinator contributions to pollen transfer is pollinator visitation activity (Ne’eman *et al.* 2010; Horsburgh *et al.* 2011; King *et al.* 2013). Pollinator visitation activity, i.e. visitation frequency or visitation rate, has been proposed as a surrogate for pollinator contribution to plant reproduction (Vázquez *et al.* 2005), but it has been proven to be a poor proxy for pollen delivery (King *et al.* 2013). However, when coupled with pollen transfer data, these findings may allow us to properly estimate pollinator contributions to pollen transfer properly (Ne’eman *et al.* 2010; King *et al.* 2013). Moreover, pollinator visitation activity is highly variable because pollinator populations vary significantly over time, especially among years (Herrera 1988). Similarly, the population of each pollinator group varies significantly over time depending on the species population dynamic, weather, and flowering plants, among other factors (Lázaro *et al.* 2010; Herrera 2019; Thomson 2019). In addition, the trends in insect, and especially pollinator decline (Hallmann *et al.* 2017, 2021), affect pollinator abundances and population dynamics (Boggs 2016). Consequently, the general decline in pollinators may have a strong impact on pollen transfer and, consequently, on plant reproductive success (Gordon *et al.* 1998; Aldercotte *et al.* 2022).

Thus, combining information about the pollen quantity transferred by individual pollinators and pollinator visitation activity, which can be measured as the visitation rate or visitation density, is crucial for estimating pollinator contribution to pollen transfer (Vázquez *et al.* 2005; Ne’eman *et al.* 2010). When multiple approaches are combined, information on pollinator contributions to pollen transfer may completely change the structure of plant–pollinator interactions compared to results that are based on only one approach (King *et al.* 2013; Ballantyne *et al.* 2015, 2017; Parker *et al.* 2016; Willmer *et al.* 2017). In addition, depending on the system, this information may reveal the unexpected importance of different pollinator taxa within the community (De Manincor *et al.* 2020; Souza *et al.* 2021; Tourbez *et al.* 2024). This may have significant consequences for our understanding of pollination systems, especially in highly generalized plant–pollinator communities, as plant–pollinator interactions show a high level of interaction turnover with time (CaraDonna *et al.* 2017; Bain *et al.* 2022). Hence, with variation in pollinator density among years (Kandori 2002; Price *et al.* 2005; Bosch *et al.* 2009; Herrera 2019) resulting in plant–pollinator interaction turnover and rewiring, the pollinator contributions to the pollen transfer of particular taxa may change significantly from year to year. Understanding the extent of variation in the contribution of pollinator taxa to pollen transfer is crucial for identifying the stability and fluctuations of pollinator importance in such systems and may help us to identify the priorities for pollination service conservation in the face of pollinator decline.

Here, we present data on the pollen loads of 35 common Central European grassland pollinator taxa, combined with a time series of plant–pollinator interactions over 11 years revealing the rate of population fluctuations for each pollinator taxon. Our goals were to quantify the variability in (i) pollen load among pollinator taxa, (ii) pollen load among individuals within pollinator taxa, (iii) flower-visitation density among pollinator taxa, and (iv) flower-visitation densities among years within pollinator taxa. We aim to provide a reliable estimation of the extent to which the contribution of each pollinator to pollen transfer is determined by their ability to carry pollen and its abundance.

## Materials and methods

### Design of the study

To investigate the contributions of different groups of pollinators to pollen transfer, we focussed on a wide spectrum of pollinators that was present at a site (see below) at the peak of flowering for two consecutive years (2020 and 2021). We measured the pollen load on the pollinator body and the proportion of conspecific pollen. We collected insect pollinators from the most abundant flowering plant species (Supplementary Material 1; Supplementary Table S1) by sampling more than 10 individuals from each pollinator group (Supplementary Table S2). To investigate interannual variations in pollinator density, we conducted pollinator and plant surveys for 11 consecutive years (2011–21). Finally, we combined information from both pollen load and pollinator density to compare the potential pollinator contribution over time.

### Study site

We conducted our research in a seminatural grassland community with a high abundance of flowering plants, called ‘K Handrkovu’, near the village of Vernýřov, Central Bohemia, Czech Republic (49.8466° N; 15.1498° E). The study site can be classified as a mosaic of moist to mesophilic and eutrophic to mesotrophic grasslands, and it hosts flowers from 50 to 60 entomogamous plant species every year (of which about 30 are sufficiently abundant to be studied) during the peak flowering period in late summer (Janovský *et al.* 2013). In this study, we combined data on pollen loads were collected during the peak of flowering activity in 2020 and 2021 (10th–20th August and 16th–21st August, respectively), and pollinator visitation data collected once a year for 11 years (2011–21) to generate plant–pollinator interaction records.

### Pollen load

**Figure 1.**
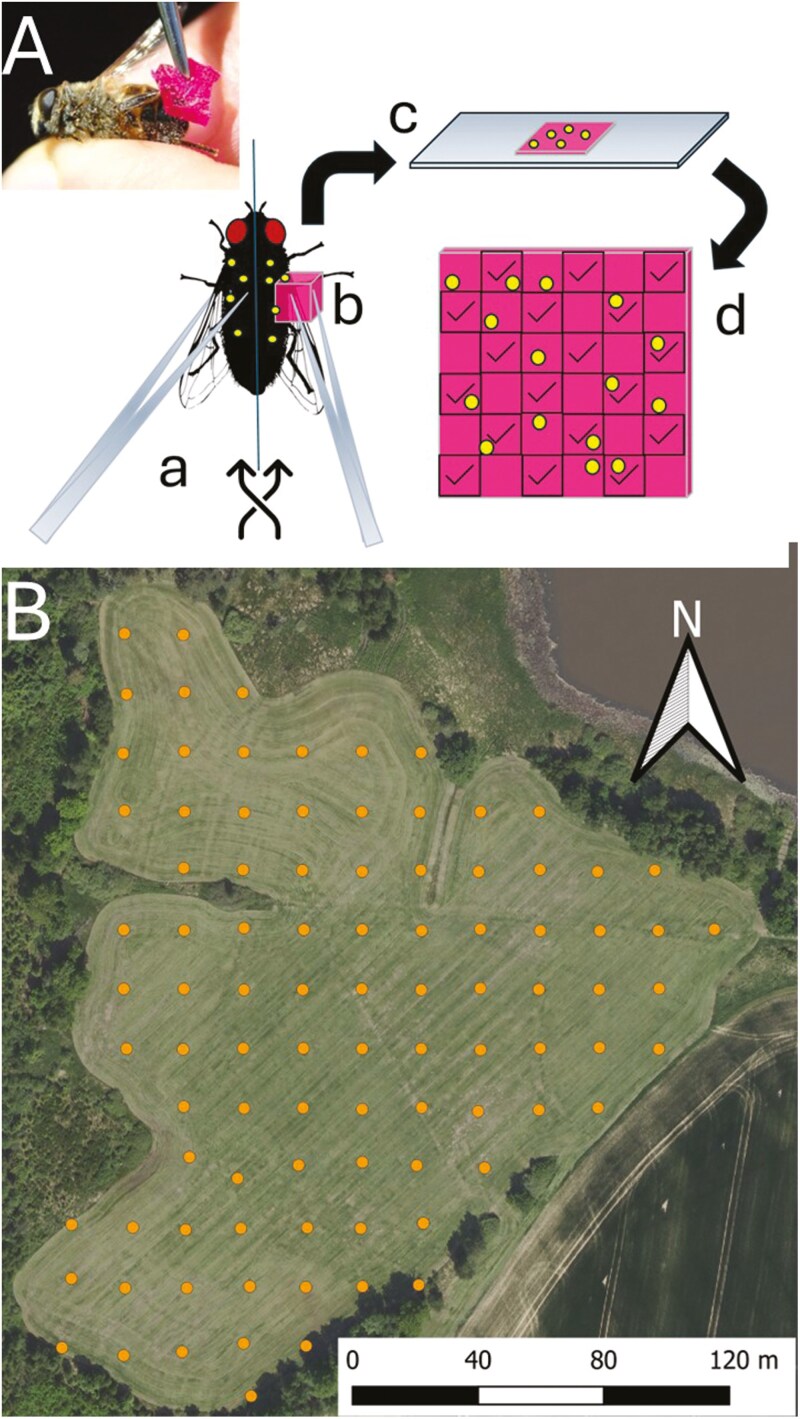
Photograph of swabbing pollen grains from pollinator’s body following the protocol established by Beattie (1971) with diagram of the method of swabbing pollen from the pollinator and the method of counting pollen grains (A). Random selection of one half of the pollinator’s body (a), Swabbing pollen grains from the half of the pollinator’s body with a small block of fuchsin jelly (b), Sample preparation on a microscope slide (c), Counting of pollen grains (dots in the diagram) using a grid and counting every second square (squares with tick marks) (d). The aerial photograph of the studied site called ‘K Handrkovu’ near Vernýřov village, Central Bohemia, Czech Republic with locations of centres of permanent plots (dots) (B).

To estimate the pollinator pollen load, i.e. the number of pollen grains carried on the body of the pollinator, we captured foraging pollinators on flowers or immediately after visits using an insect net. We identified pollinators and the visited plants. We killed pollinator individuals immediately after capture and swabbed pollen grains from one half (randomly selecting left or right) of each pollinator’s body (including mouth parts, such as proboscis) with a small block of fuchsin jelly (see Fig. 1) as in Beattie (1971). For bees and bumblebees, we did not sample the pollen that was packed in pollen baskets because this pollen cannot participate any further in pollination. On the same day, we produced semi-permanent samples by melting fuchsine jelly blocks with a candle on a microscope slide and sealed with nail polish. Later, we counted and determined the pollen grain types under a light microscope. Using a fine grid (1 × 1 mm), we divided the sample into squares and counted the pollen grains in every second square (Fig. 1A). In each sample, we scored the categories on each second square on a semi-quantitative scale based on the number of all pollen grains (e.g. 0, 1, 2–10, 11–25, and 26 + pollen grains in the square). As a result, we identified 200 randomly chosen individual pollen grains in each sample (if there were fewer grains, all of them were identified) based on their morphology and a reference collection obtained from the study site by collecting pollen grains from anthers of all flowering plant species using fuchsine-stained cubes of glycerine jelly. For each sample, we calculated the proportion of pollen from plants visited by pollinators (conspecific pollen) and the proportion of pollen from all other plant species (heterospecific pollen). The final amount of pollen grains thus corresponded to the number of pollen grains present on one quarter of the individual pollinator bodies, because we obtained a sample from half of the pollinator’s body wiped with jelly in which we counted half of the pollen grains (see a scheme Fig. 1A).

### Pollinator visitation activity

We followed Janovský *et al.* (2013) to collect data on pollinator visitation activity. In short, 93 plots with sizes of 4 × 4 m² each were placed in the centre of each grid point of a 20 × 20 m² grid covering a total area of about 300 × 300 m² of the study site. We surveyed each plot to record all interactions between flower-visiting pollinators and flowering plants. During surveys, all flowers within the plot were checked exactly one time to avoid pseudo-replications and all interactions occurring at the moment of the survey were recorded. The survey was not time-limited to avoid potential bias by the researcher’s ability to record all the interactions in a limited time. However, the majority of the surveys were shorter than 5 min. Pollinator surveys were conducted for each plot at least twenty times at the peak of flowering (mid-August) each year, and all pollinators visiting the flowers and touching the reproductive organs of the plants were recorded. Each pollinator individual was recorded only once. Counts were conducted during the activity period of most pollinators (7:00 to 19:00), with observations randomized across plots, days, and times of the day. Nocturnal and crepuscular pollinators were not included in the observations. Each recorded pollinator was identified to the lowest possible taxonomic level in the field and the time of day and visited plant species were recorded. From the pollinator counts, we calculated the final visitation density (i.e. number of visits per plot divided by the number of surveys of individual plots conducted in the particular year) of each pollinator group over the entire collection time.

### Pollinators

We focussed on the wide spectrum of pollinators that were present at the study site. The captured pollinators were identified to the lowest possible taxonomic level with the help of specialists from the Department of Zoology, Faculty of Science, Charles University in Prague, and divided into pollinator groups (Table 1) based on their taxonomy, morphology, similarity in functional traits and ability to identify individual taxa. Some groups contain only one species of pollinator (*Apis*, *Bombus lap.* or *Eristalis ten*.), while other groups are composed of several species of the same genus (*Lucilia*, *Mordella*, or *Syrphus*) or family (Halictidae or Tenthredinidae). Several groups (Tachinidae and Small Tachinidae) are composed of several different species with similar ecology and morphology that cannot be easily distinguished (due to high similarity in their traits). Because identification of pollinators in the field is more difficult, the pollinator groups that are used in pollinator density surveys are slightly different and less specific than the pollinator groups that are used in pollen load surveys.

### Plants

We focussed on the most abundant and the most visited flowering plant species at the study site. Abundances of plants were measured as the presence/absence of flowering stalks in each subplot (0.5 × 0.5 m²) of each permanent plot (4 × 4 m²; see Janovský *et al.* 2013) (Supplementary Table S1). The majority of plant species included in this study are common and well-recognized in our area, although for some plant species, we used a botanical identification key (Kaplan *et al.* 2019).

### Data analysis

Based on the data obtained from the plant and pollinator surveys, we calculated pollinator abundances of the target pollinator taxa for each year between 2012 and 2021. We standardized the data for the recorded plant–pollinator interactions per plot in each year with the number of surveys of individual plots conducted in the particular year. Then, we summed the interactions from all plant species for each pollinator taxon to obtain an estimate of the pollinator abundances in all plots for each year. The final variable then represents the sum of the pollinator interactions for each survey in a particular year. We counted the number of pollen grains on one quarter of the pollinator bodies, which was used as a dependent variable in the analysis of variance (ANOVA). The group of pollinators was the independent variable. We log-transformed the dependent variables.

We generated a data subset using only the plant species for which we collected a sufficient number of pollinators (more than five individuals) from different functional pollinator groups. We used data from *Selinum carvifolia*, *Ranunculus* sp., *Daucus carota*, *Centaurea jacea*, *Succisa pratensis*, *Potentilla* sp., *Pimpinela saxifraga*, *Lathyrus pratensis*, *Prunella vulgaris*, *Lythrum salicaria*, and *Sanguisorba officinalis*. We used a quasi-binomial generalized linear model (GLM) (Crawley 2007) to determine the proportion of conspecific and heterospecific pollen. We generated ranks of pollen loads and proportions of conspecific pollen for all pollinator taxa to compare them. We used Spearman rank correlation analysis to test the presence of a correlation between pollen load and the proportion of conspecific pollen.

Finally, to determine the relative variability in the amount of pollen carried within the groups, we calculated the coefficient of variation (CV = standard deviation σ/mean μ) of each pollinator group. To compare the relative variability in pollen load among the pollinator groups, we calculated the CV of the average number of pollen grains carried by each pollinator group.

## Results

We captured 618 individual pollinators from a wide spectrum of taxa that together carried more than 800 000 pollen grains on their bodies (an estimation of the sum of all counted pollen grains). On average, each pollinator carried 1100 pollen grains. To determine the differences among pollinators, we sorted them into 31 taxonomic pollinator groups (Table 1) and measured the number of pollen grains carried on their bodies and the proportion of conspecific pollen. In total, we recorded 45 472 individual visits of flowers by pollinators over 11 years.

### Pollen load

**Figure 2.**
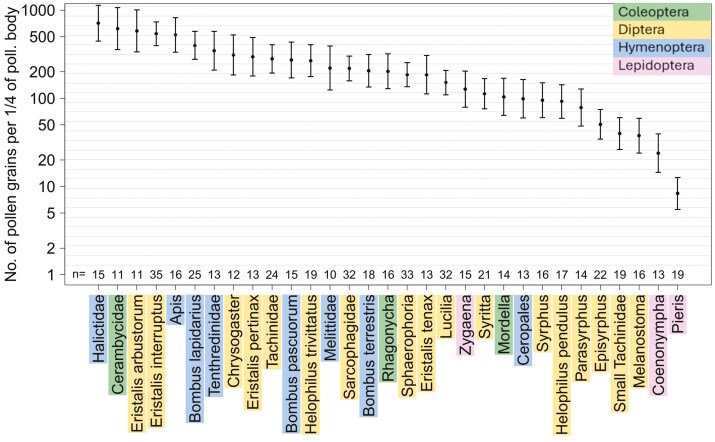
Ranked pollen loads, i.e. number of pollen grains carried on the body, among pollinator groups, studied in flowering seasons 2020 and 2021. Pollen load is expressed as a log value of the number of pollen grains carried on a quarter of the pollinator’s body. Error bars indicate the 95% confidence intervals of the estimate of the mean values. Numbers under error bars represent the number of sampled individuals of pollinator groups.

**Figure 3.**
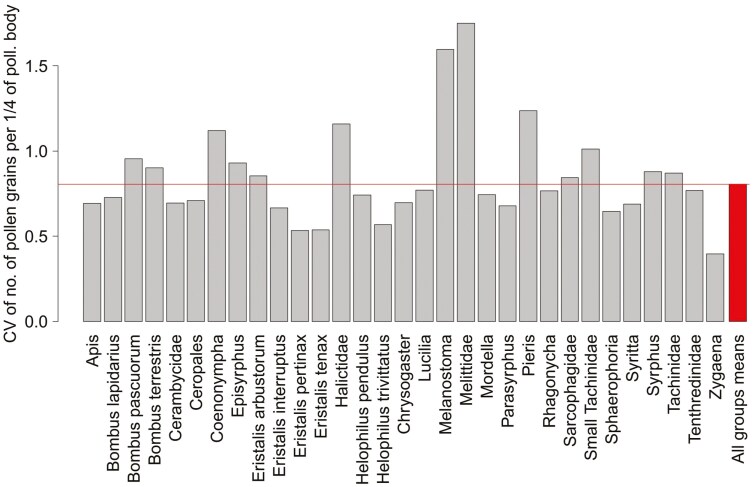
Coefficient of variation (standard deviation/mean) of carried pollen load for each of pollinator groups showing relative variability within groups. Coefficient of variation for all groups mean pollen load values (the last bar) showing relative variability among groups.

Pollen load, i.e. the amount of pollen carried on the pollinator bodies, significantly differed among pollinator groups (*F*_30, 531_ = 21.3; *P* < .001; ANOVA), explaining 54.6% of the total variability. Halictidae, Cerambycidae, and *Eristalis arbustorum* tended to have the greatest pollen load, but these loads were not very different from those of the other groups (Fig. 2). In contrast, *Pieris* carried the smallest number of pollen grains. Overall, the Hymenoptera carried more pollen than other orders, and the Lepidoptera carried the least amount of pollen. In general, the significant differences are only due to some groups with a very large or very small amount of pollen load (Fig. 2). The coefficients of variation for the pollen loads carried on pollinator bodies showed that the variability within pollinator groups was similar to that among groups for most pollinator groups (Fig. 3). Some pollinator taxa, such as *Melanostoma* or *Pieris*, were more variable within the group than among the pollinator groups. In contrast, the pollen loads of pollinators such as *Zygaena*, *Eristalis pertinax*, and *Apis* were more consistent within the groups than among the other pollinator groups (Fig. 3).

### Proportion of conspecific pollen

**Figure 4.**
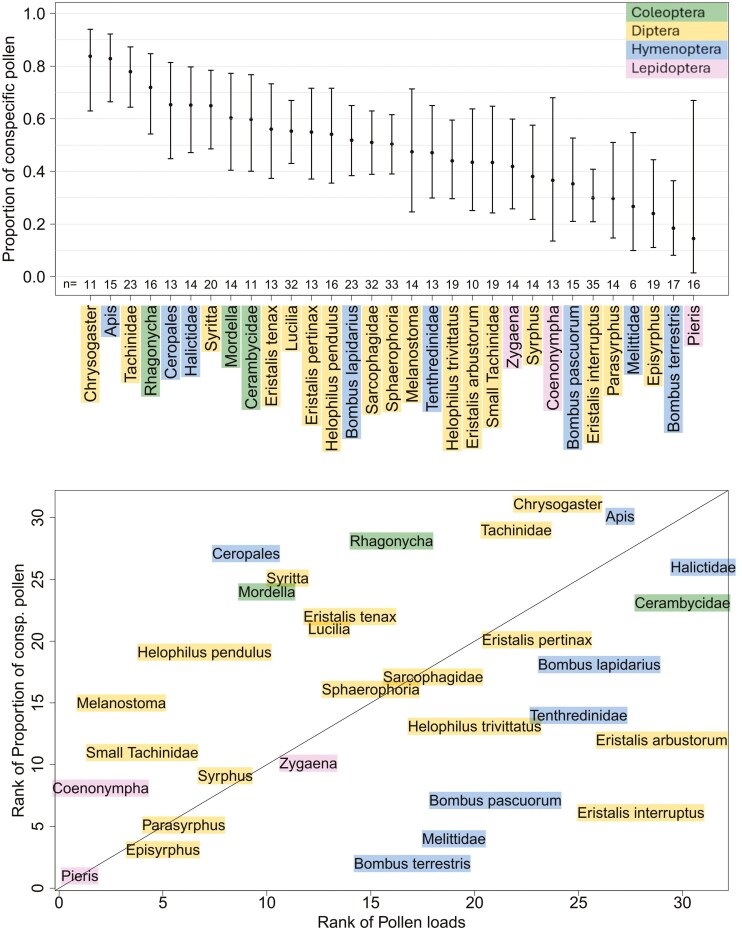
(A) Proportion of conspecific pollen (pollen of the plant on which the pollinator was caught) carried on pollinator’s body belonging to pollinator groups in flowering seasons 2020 and 2021. Error bars indicated the standard errors of the expected proportion of conspecific pollen. Numbers under error bars represent numbers of individuals from pollinator groups from sufficient abundant flowering plants. (B) Comparison of results from conspecific pollen proportion model and pollen load model. A higher rank value of conspecific pollen proportion model means higher proportion of conspecific pollen carried on the pollinator’s body. A higher rank value of pollen load model means more pollen grains carried on the pollinator’s body.

The proportion of conspecific pollen carried on the pollinator bodies significantly differed among pollinator groups (*df* = 30; deviance = 9864.2; *F* = 3.91; *P* < .001; GLM with *F* test) (Fig. 4A). The residual deviance of the model is 49.515, with 505 residual degrees of freedom. *Chrysogaster*, followed by *Apis*, carried the highest proportion of conspecific pollen, whereas *Pieris* carried the lowest one. In general, the Lepidoptera carried a lower proportion of conspecific pollen than the other orders of pollinators, but differences are due to only those between pollinator groups with the highest and lowest proportions (Fig. 4A). After comparing the results from the pollen load model and the proportion of the conspecific pollen model, we found that some taxa, such as *Apis*, Halictidae, *Chrysogaster*, and *Cerambycidae*, carried large amounts of pollen with high proportions of conspecific pollen grains. In contrast, other pollinator taxa, such as *Pieris*, *Coenonympha*, *Episyrphus*, and *Parasyrphus*, carried small amounts of pollen grains with a low proportion of conspecific pollen grains (Fig. 4B). Pollinators such as *Eristalis interruptus* or *Eristalis arbustorum* carried large amounts of pollen but with a low proportion of conspecific pollen, and pollinators such as *Ceropales*, *Syritta*, or *Mordella* carried small amounts of pollen grains but with a high proportion of conspecific pollen (Fig. 4B). We found a moderate positive relationship between pollen loads and proportions of conspecific pollen carried by pollinators (rho = 0.35; *S* = 3216; *P* = .053; Spearman rank correlation).

### Density of pollinator-flower interactions

**Figure 5.**
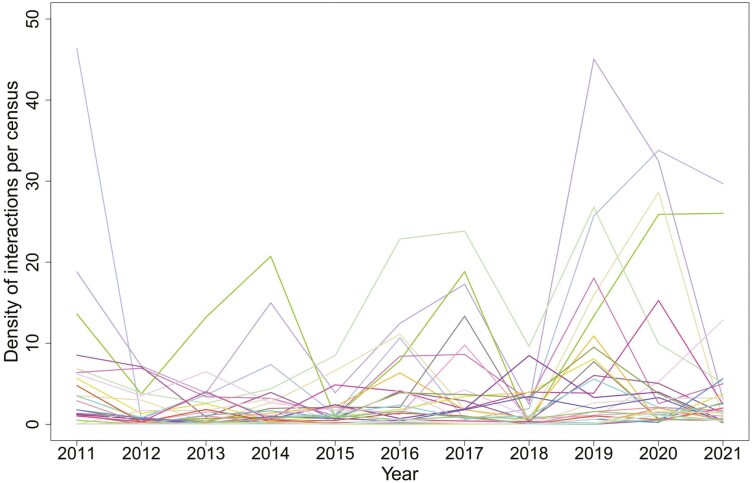
Variation in density of interactions of individual pollinator groups (the sum of pollinator interactions per survey of permanent plots) over the years 2011–2021.

**Figure 6.**
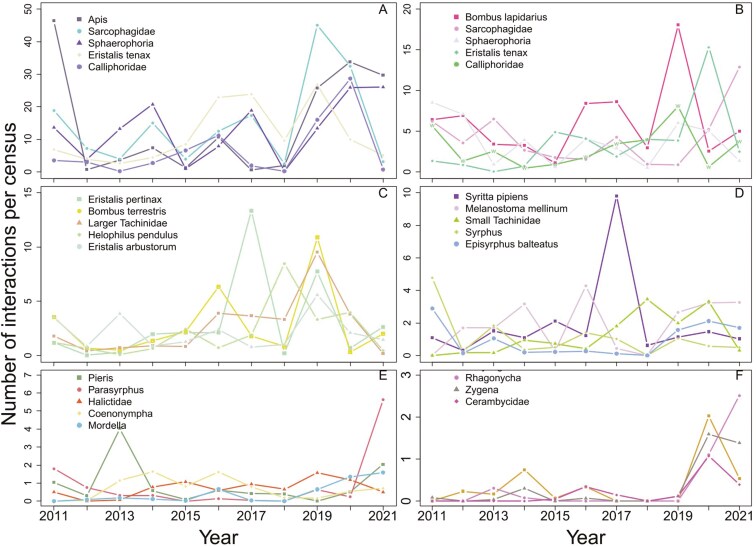
Changes in pollinator group interaction density per survey of permanent plots over years (2011–2021). Pollinator groups are grouped according to the mean number of interactions per census (A, B, C, D, E, F).

The density of pollinator-flower interactions differed among the pollinator groups. On average across all years, we observed the highest density for pollinator groups such as Sarcophagidae, *Sphaerophoria*, and *Eristalis tenax*. In contrast, the lowest density values were observed for *Zygaena*, *Rhagonycha*, and *Chrysogaster*. The interaction densities of most pollinator groups changed significantly over the years (Figs. 5 and 6). The density of pollinator-flower interactions of most pollinator groups varied over the years (Figs. 5 and 6), in particular in the Sarcophagidae, *Lucilia*, *Sphaerophoria*, and *Apis mellifera* groups.

## Discussion

The main goal of our paper was to investigate the contribution of different groups of pollinators to pollen transfer and to provide an estimation of the extent to which the contribution of each pollinator is determined by their ability to carry pollen and its abundance. We combined data on the pollen loads of 35 common Central European grassland pollinator taxa with a time series of plant–pollinator interactions over 11 years revealing the rate of their population fluctuations. Our results showed low interspecific differences in the pollen loads carried by various groups of flower-visiting insects, i.e. the pollinator taxa carried pollen amounts that were more similar than expected (Fig. 2). Pollen loads ranged from single units of pollen grains to thousands of pollen grains per individual pollinator, variability mainly accounted for by intraspecific differences among individual pollinators (Fig. 2).

Our results revealed notable variability in pollen load among individual pollinators. Morphological and behavioural traits are often suggested as key determinants of the amount and quality of pollen carried, with factors such as body size (Földesi *et al.* 2021), and hairiness (Stavert *et al.* 2016) shown to influence pollen load. Although in our study we did not focus on a direct comparison of pollinator traits per se, the comparison of the total amount of pollen carried by different pollinator taxa indicated that the generally larger and more hairy taxa, such as bees and large hoverflies (*Eristalis*), carried more pollen than smaller pollinators (Fig. 2).

Overall, we found no difference between the pollen load of Diptera and Hymenoptera, or between that of bees and hoverflies, as the members of each group covered the whole range, from carrying a small amount of pollen to the biggest amount sampled in our study (Fig. 2). Other studies have shown that large hoverflies may carry as much pollen as bees of similar size (Mahy *et al.* 1998), which is also supported by our results (Fig. 2). Similarly, we found a wide range of pollen loads was found on beetles, with, on the one hand, Mordellidae, carrying only a low amount of pollen and, on the other hand, Cerambycidae carrying a high amount of pollen. Only Lepidoptera was consistently found to be relatively fewer effective pollinators (Figs. 2 and 4A), which could be caused by a limited number of species (we did not record nocturnal Lepidoptera, which are assumed to be effective pollinators; Walton *et al.* 2020; Ribas-Marquès *et al.* 2022). Although our data suggested that Lepidoptera (represented by butterflies and *Zygaena* moths) have relatively low pollen load, in the case of *Zygaena*, their low pollen load may be caused by their occurrence on flowers of species that produced low amounts of pollen. Thus, they are likely important pollinators, particularly for those plants that are not attractive to other pollinators and have a restricting flower shape targeting specific pollinator traits. The undeniable advantage of Lepidoptera pollinators is the longer flight distance, which can lead to a higher rate of outcrossing in visited plants (Herrera 1987).

Moreover, the total amount of pollen carried on pollinator body can be misleading when comparing pollinator contributions to pollen transfer within a diverse community due to the interspecific differences in the amount of produced and presented pollen (Cruden 2000; Gong and Huang 2014) and the accessibility of flowers for particular pollinators (Stang *et al.* 2009). Further research, coupled with proper estimates of pollen production and pollinator foraging bouts, are necessary to understand further the contribution of pollinators to pollen transfer among plant species.

The proportion of conspecific pollen in the pollen load varied considerably among pollinators from 20% to 80% of the conspecific pollen grains in the pollen load (Fig. 3). This high variation can be explained by the difference in foraging behaviour, namely, flower constancy (Waser 1986) and a high degree of specialization, as well as grooming behaviour of some pollinators (Koch *et al.* 2017). Flower constant pollinators tend to visit the same plant species during subsequent flights, resulting in greater proportions of conspecific pollen on the pollinator body. Our results showed a greater proportion of conspecific pollen in honeybees that are known for their flower constancy (Hill *et al.* 1997; Grüter *et al.* 2011) and, in *Chrysogaster solstialis*, a small hoverfly that, in our system, predominantly visits only a narrow set of plant species, mainly from the Apiaceae family (unpublished data). Bumblebees, especially *Bombus terrestris*, carried only approximately 50% of conspecific pollen grains, which may be caused by their simultaneous visitations of at least two species during foraging bouts, a type of behaviour known as majoring and minoring foraging (Heinrich 1979; Chittka *et al.* 1997). It is also important to note the high intraspecific variation in the pollen composition on the pollinator body, which may indicate the high importance of individual behaviour to the composition of pollen load. We did not track the previous visits of pollinators, and thus, we cannot address the relationship between pollinator foraging behaviour and pollen composition on pollinators’ body.

In contrast to the low difference in total pollen amount carried by pollinator taxa, the plant–pollinator interaction abundances varied considerably over the years and among pollinators (Figs. 5 and 6). The high variation in pollinator abundance is not surprising and may be caused by several mechanisms, such as climatic conditions affecting both pollinator populations and foraging behaviour (Forrest 2015) and the amount and species composition of flowering plants at the study site and in the surrounding area across the study period. Moreover, the variation in honeybee abundance can be explained by the differences in the spatial distribution of managed bee hives in the surrounding area (Guzman *et al.* 2019).

Interannual climate variation can profoundly impact insect populations (Boggs 2016) and, consequently, the structure of the plant–pollinator network (Fang *et al.* 2024). Our study system was subjected to a period of drought between 2015 and 2018 (Moravec *et al.* 2021), which may be responsible for the decreased number of recorded interactions within this period. However, the high variation in pollinator species abundances may not necessarily result in changes in the pollination network’s general parameters, including, connectance, nestedness, and degree of generalization (DuPont *et al.* 2009).

When considering how the ability to carry pollen and pollinator abundance affect the pollinator contribution to pollen transfer, we conclude that in our study system, the interannual differences in pollinator abundance may play a major role in driving the pollen transfer pattern. Consequently, due to changes in abundance over time, plants may experience a high degree of turnover of the pollinator spectral composition (DuPont *et al.* 2009; CaraDonna *et al.* 2017).

From an evolutionary perspective, the proportionally greater variation in pollinator abundance than in pollen load may place plant species under strong pressure to adopt a generalized strategy to ensure their pollination despite the unpredictability of pollinator abundance and composition (Waser *et al.* 1996; Ohashi *et al.* 2021). A recent study from a highly stochastic alpine environment revealed only moderate interannual variation in the amount and composition of pollen deposited on stigmas (Fang *et al.* 2019), suggesting that the plant species within the community may be able to adapt to dynamic changes in pollinator composition (Frachon *et al.* 2023). Conversely, changes in plant species composition can affect pollinator populations as their food sources change so pollinators must adapt to new conditions (Herrera 2019; Thomson 2019). Further research focussing on the direct effect of pollinator and plant turnover on pollen transfer are necessary to provide a deeper insight into the ecological and evolutionary pressures on plant adaptation strategies to secure pollen transfer. In addition, we need to keep in mind that pollen transfer is only one part of the plant life cycle, and that other processes, such as pollinator-mediated seed production or seed germination, may affect plant fitness and response to climate changes.

## Conclusions

We found that the pollen amount carried on the pollinator body varied among pollinator groups, but the differences were small, and that such variability was mainly driven by intraspecific differences among individual pollinators. In contrast, the abundance of pollinators fluctuated highly from year to year, being presumably responsible for the majority of the variation in the species-species importance of pollinators for a given plant species. In general, pollinator contribution to pollen transfer in our system depended on the amount of pollen carried and the number of plant–pollinator interactions, which varied over the years. The high variation in pollinator abundance over time may lead to a high turnover in plant–pollinator interactions and pollen transfer, with changes in the importance of individual pollinators. Thus, pollinator diversity may be crucial in compensating for the variation in pollinator abundance at the community level, especially in the context of the current insect and pollinator declines in several ecosystems.

## Supplementary Material

plaf009_suppl_Supplementary_Materials_1_Tables_S1_Figures_S1-S2

## Data Availability

All data can be found in the Figshare repository: https://doi.org/10.6084/m9.figshare.27933357.v1
