# Supplementary material for: Interannual differences in pollinator contributions to pollen transfer are mainly driven by changes in pollinator abundance
Source: AoB Plants. 2025 Feb 22;17(2):plaf009. doi: 10.1093/aobpla/plaf009 (PMC12000867; doi:10.1093/aobpla/plaf009)
Supplement: plaf009_suppl_Supplementary_Materials_1_Tables_S1_Figures_S1-S2 [file plaf009_suppl_supplementary_materials_1_tables_s1_figures_s1-s2.zip › freudenfeld_supplement_final.pdf]

Interannual differences in pollinator contributions to pollen transfer are mainly driven by changes in pollinator abundance

Martin Freudenfeld[1]*, Jakub Štenc[1,7], Jiří Hadrava[2], Michael Mikát[2], Eva Matoušková[2], Klára Daňková[2], Tomáš Jor[2], Tadeáš Ryšan[2], Klára Koupilová[3], Jan Simon-Pražák[2,4], Tomáš Dvořák[5], Zdeněk Janovský[6]

[1] Department of Botany, Faculty of Science, Charles University, Benátská 2, 128 41 Prague, Czech Republic

[2] Department of Zoology, Faculty of Science, Charles University, Viničná 7, 128 41 Prague, Czech Republic

[3] Rovná 1333, Sulice, 25168, Czech Republic

[4] Museum of Eastern Bohemia in Hradec Králové, Eliščino nábřeží 465, 500 03 Hradec Králové 3, Czech Republic

[5] Správa Národního parku Podyjí, Na Vyhlídce 5, 669 02 Znojmo

[6] Svatý Jan t. Krsovice 1, 285 04 Uhlířské Janovice, Czech Republic

[7] Department of Population Biology, Institute of Botany, Czech Academy of Sciences, Lesní 322, 25243 Průhonice, Czech Republic

*Corresponding author, e-mail address: martinf13@seznam.cz

Supplementary Information

Table S1. Absolute (abs.) and relative (rel.) abundances of flowering plant species in 93 permanent plots in the locality K Handrkovu in 2020 and 2021. Abundances were measured as presence/absence of flowering stalks in each subplot (0.5 × 0.5m) of each permanent plot (4 × 4m) (see Janovský et al. 2013).

Plant species	2020 abs.	2021 abs.	2020 rel.	2021 rel.
Plantago lanceolata	3053	2569	18.3 %	11.5 %
Ranunculus acris	2505	3013	15.0 %	13.5 %
Prunella vulgaris	2487	2383	14.9 %	10.6 %
Centaurea jacea	952	1883	5.7 %	8.4 %
Potentilla erecta	1164	1440	7.0 %	6.4 %
Lathyrus pratensis	465	1628	2.8 %	7.3 %
Lotus corniculatus	601	1114	3.6 %	5.0 %
Pimpinella saxifraga	692	894	4.2 %	4.0 %
Trifolium repens	342	1156	2.1 %	5.2 %
Galium album	1331	67	8.0 %	0.3 %
Ranunculus flammula	446	788	2.7 %	3.5 %
Sanguisorba officinalis	490	722	2.9 %	3.2 %
Trifolium pratense	339	596	2.0 %	2.7 %
Trifolium hybridum	328	560	2.0 %	2.5 %
Achillea ptarmica	5	728	0.0 %	3.3 %
Selinum carvifolia	248	417	1.5 %	1.9 %
Succisa pratensis	122	253	0.7 %	1.1 %
Other species in total	1097	2166	6.6 %	9.7 %

Table S2. Pollinator taxa sampled for pollen loads, number of samples, and their classification into pollinator groups. Each sampled pollinator was identified to order, family, and pollinator group.

Order	Family	Pollinator	Number of samples	Group
Coleoptera	Cantharidae	*Rhagonycha fulva*	16	Rhagonycha
	Cerambycidae	*Stenurella melanura*	9	Cerambycidae
		Stictoleptura rubra	2	Cerambycidae
	Mordellidae	*Mordella* sp.	14	Mordella
Diptera	Calliphoridae	*Lucilia* sp.	32	Lucilia
	Sarcophagidae	Sarcophagidae	32	Sarcophagidae
		Episyrphus balteatus	22	Episyrphus
		Eristalis arbustorum	11	Eristalis arb.
		Eristalis interruptus	35	Eristalis int.
		Eristalis pertinax	13	Eristalis per.
		Eristalis tenax	13	Eristalis ten.
		Helophilus pendulus	17	Helophilus pen.
		Helophilus trivittatus	19	Helophilus tri.
		Chrysogaster sp.	12	Chrysogaster
		Melanostoma mellinum	16	Melanostoma
		Parasyrphus sp.	14	Parasyrphus
		Sphaerophoria scripta	33	Sphaerophoria
		Syritta pipiens	21	Syritta
		Syrphus sp.	16	Syrphus
	Tachinidae	*Eriothrix* sp.	2	Tachinidae
		Phasiinae	22	Tachinidae
		small Tachinidae	19	Small Tachinidae
	Apidae	*Apis mellifera*	16	Apis
		Bombus lapidarius	25	Bombus lap.
		Bombus pascuorum	15	Bombus pas.
		Bombus terrestris	18	Bombus ter.
	Halictidae	*Halictus tumulorum*	1	Halictidae
		Lasioglossum leucozonium	1	Halictidae
		Lasioglossum politum	1	Halictidae
		Lasioglossum quadrinotatum	3	Halictidae
		Lasioglossum villosulum	1	Halictidae
		Lasioglossum sp.	8	Halictidae
	Melittidae	*Macropis europaea*	5	Melittidae
		Melitta nigricans	5	Melittidae
	Pompilidae	*Ceropales maculata*	12	Ceropales
		Ceropales pygmaea	1	Ceropales
	Tenthredinidae	*Athalia rosae*	12	Tenthredinidae
		Tenthredo sp.	1	Tenthredinidae
Lepidoptera	Nymphalidae	*Coenonympha pamphilus*	13	Coenonympha
	Pieridae	*Pieris rapae*	19	Pieris
	Zygaenidae	*Zygaena* sp.	15	Zygaena
**All samples**			**562**	

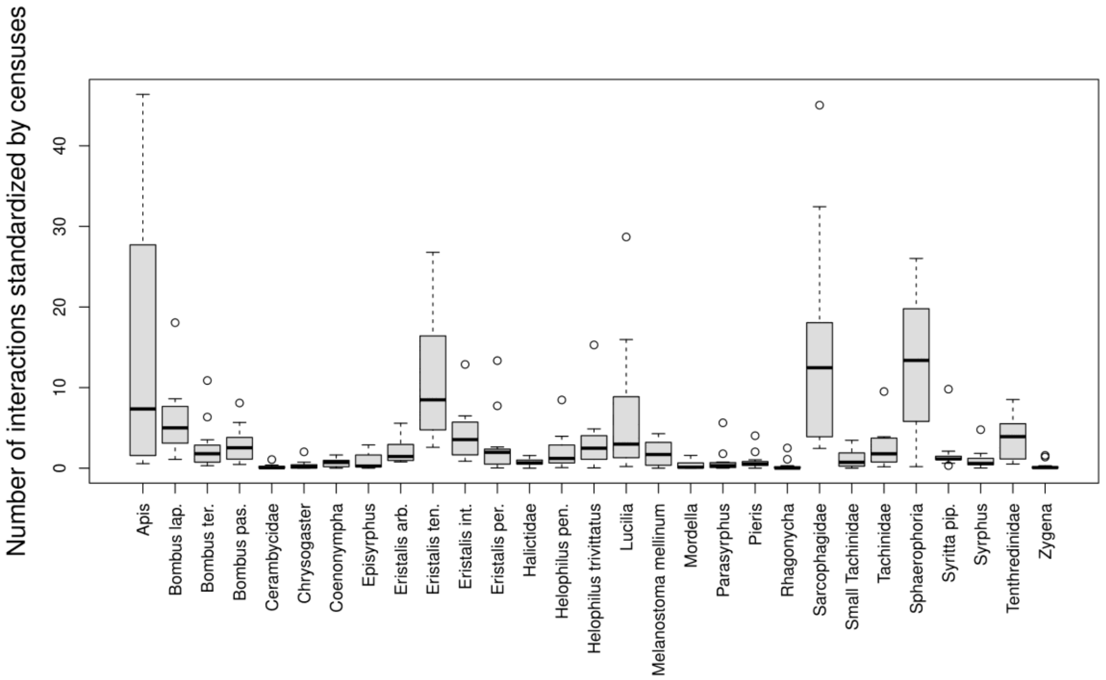

Figure S1. Average number of interactions of all pollinator groups for the years 2011 to 2021 standardized by censuses of permanent plots. Methods of collecting data described in detail in Janovský et al. 2013.

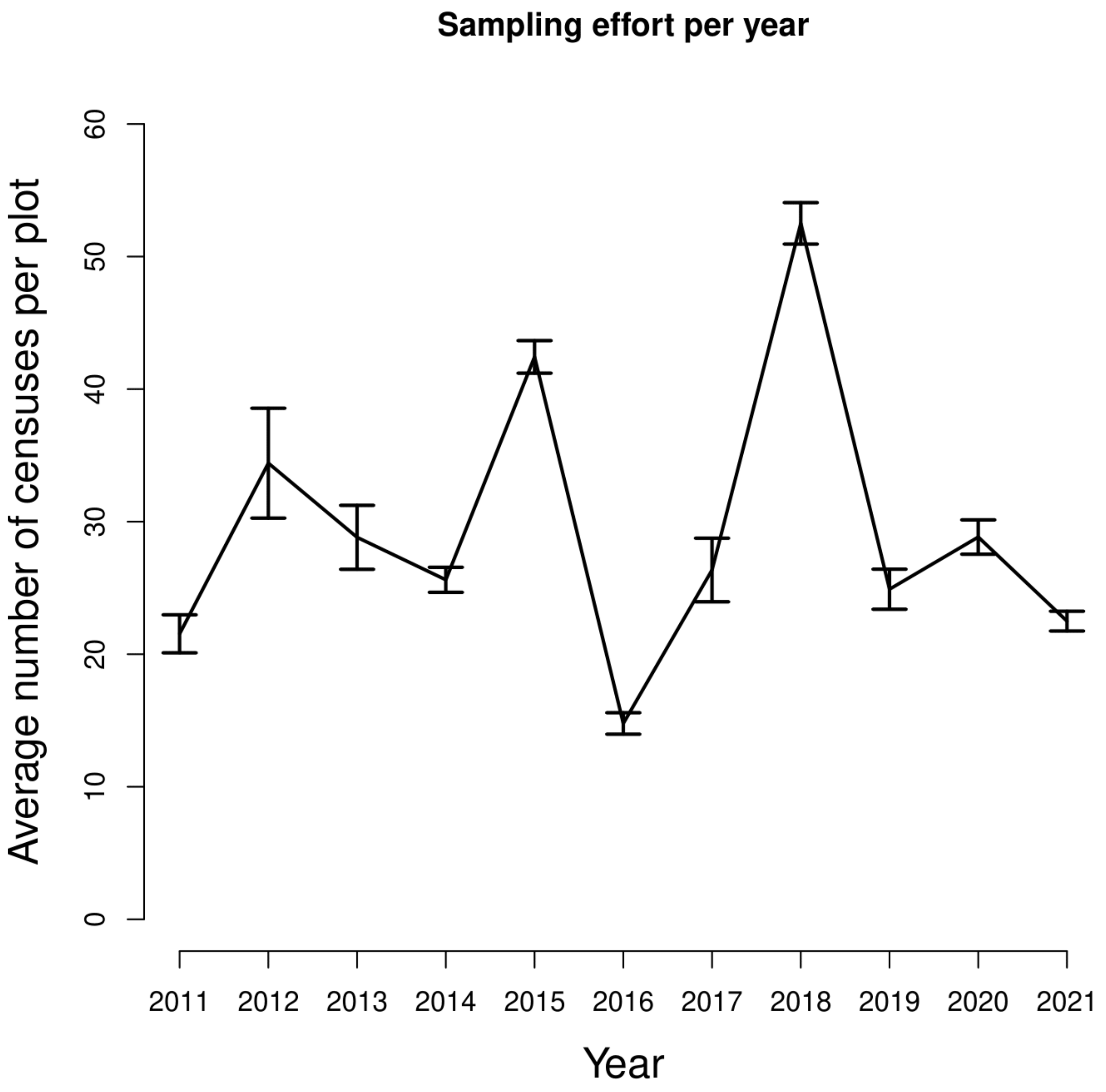

Figure S 2. Sampling effort i.e., average number of pollinator censuses per plot over years.

Literature Cited

Janovský Z, Mikát M, Hadrava J, *et al.* 2013. Conspecific and Heterospecific Plant Densities at Small-Scale Can Drive Plant-Pollinator Interactions. *PLoS One* **8**(10): e77361. https://doi.org/10.1371/journal.pone.0077361
