# Supplementary figures and images for: Interannual differences in pollinator contributions to pollen transfer are mainly driven by changes in pollinator abundance
Source: AoB Plants. 2025 Feb 22;17(2):plaf009. doi: 10.1093/aobpla/plaf009 (PMC12000867; doi:10.1093/aobpla/plaf009)

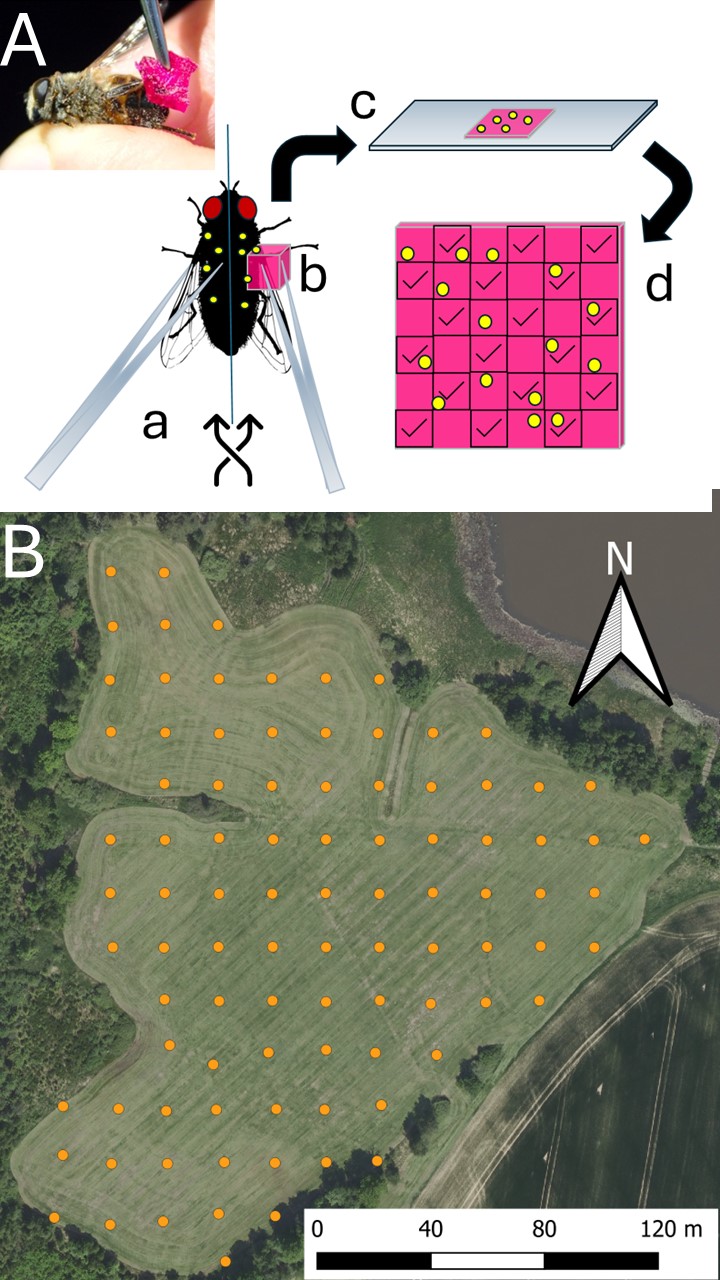

Supplement: plaf009_suppl_Supplementary_Materials_1_Tables_S1_Figures_S1-S2 [file plaf009_suppl_supplementary_materials_1_tables_s1_figures_s1-s2.zip › aobplants-24089S2R1-f01-z-4c.jpg]

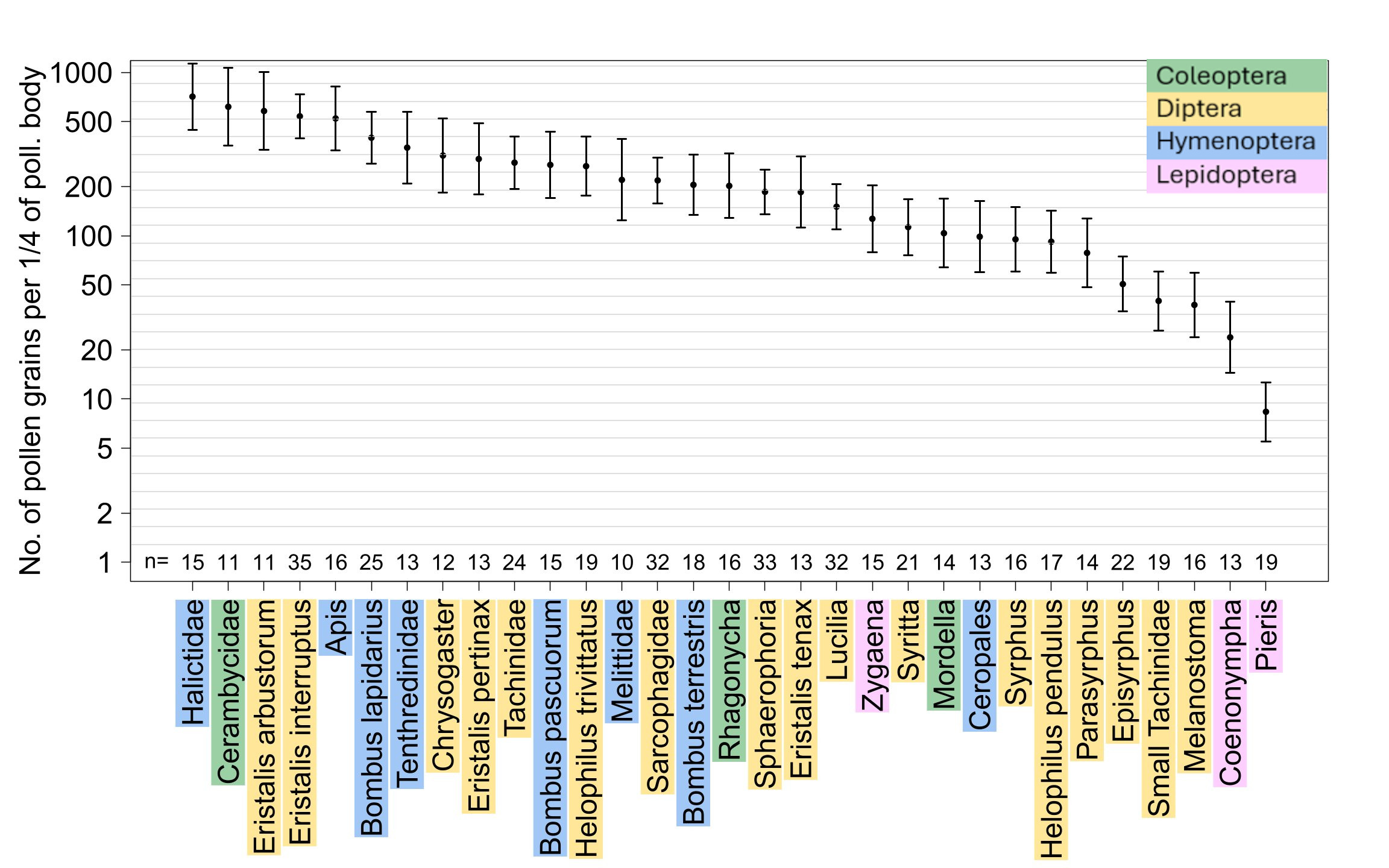

Supplement: plaf009_suppl_Supplementary_Materials_1_Tables_S1_Figures_S1-S2 [file plaf009_suppl_supplementary_materials_1_tables_s1_figures_s1-s2.zip › aobplants-24089S2R1-f02-z-4c.jpg]

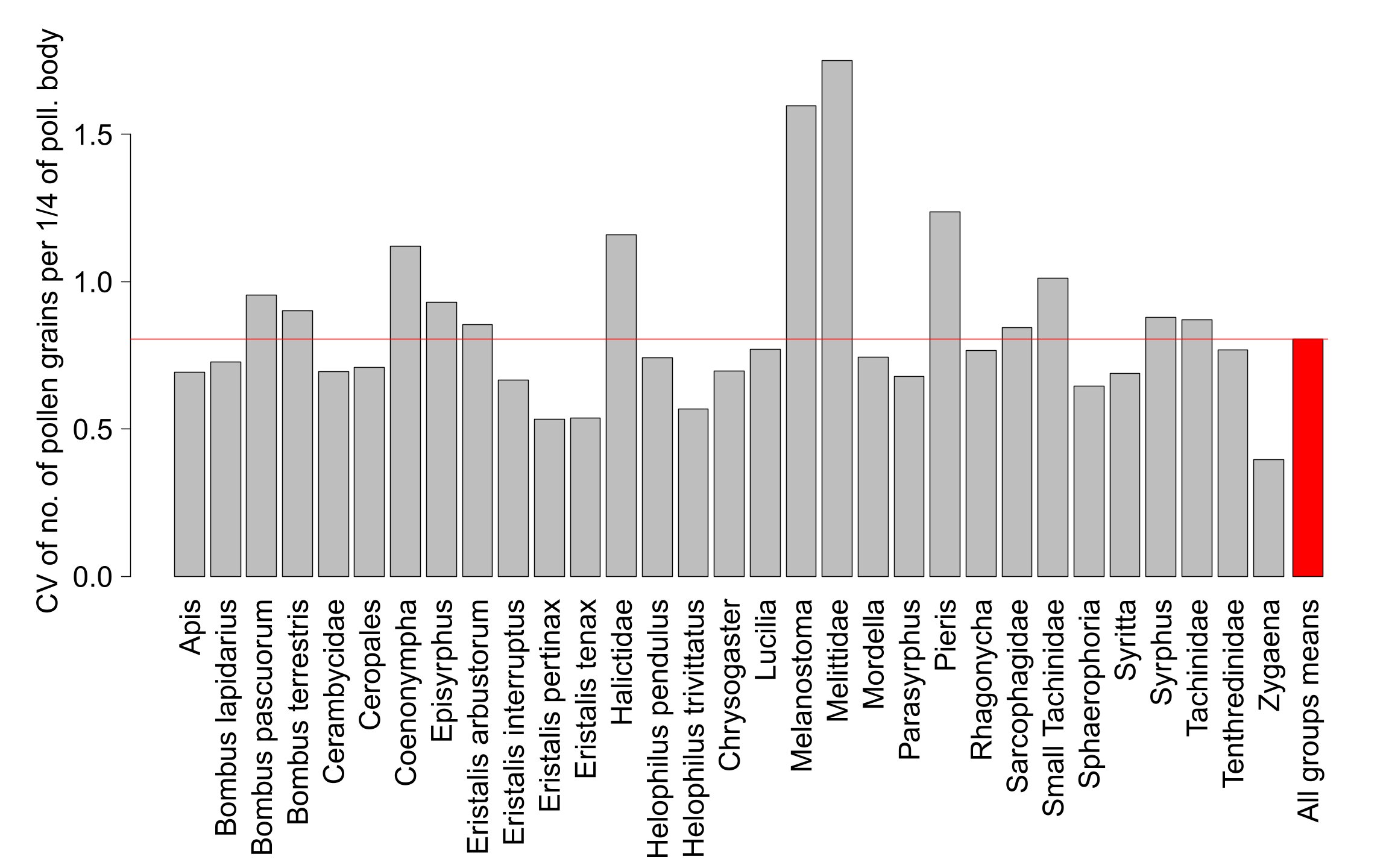

Supplement: plaf009_suppl_Supplementary_Materials_1_Tables_S1_Figures_S1-S2 [file plaf009_suppl_supplementary_materials_1_tables_s1_figures_s1-s2.zip › aobplants-24089S2R1-f03-z-4c.jpg]

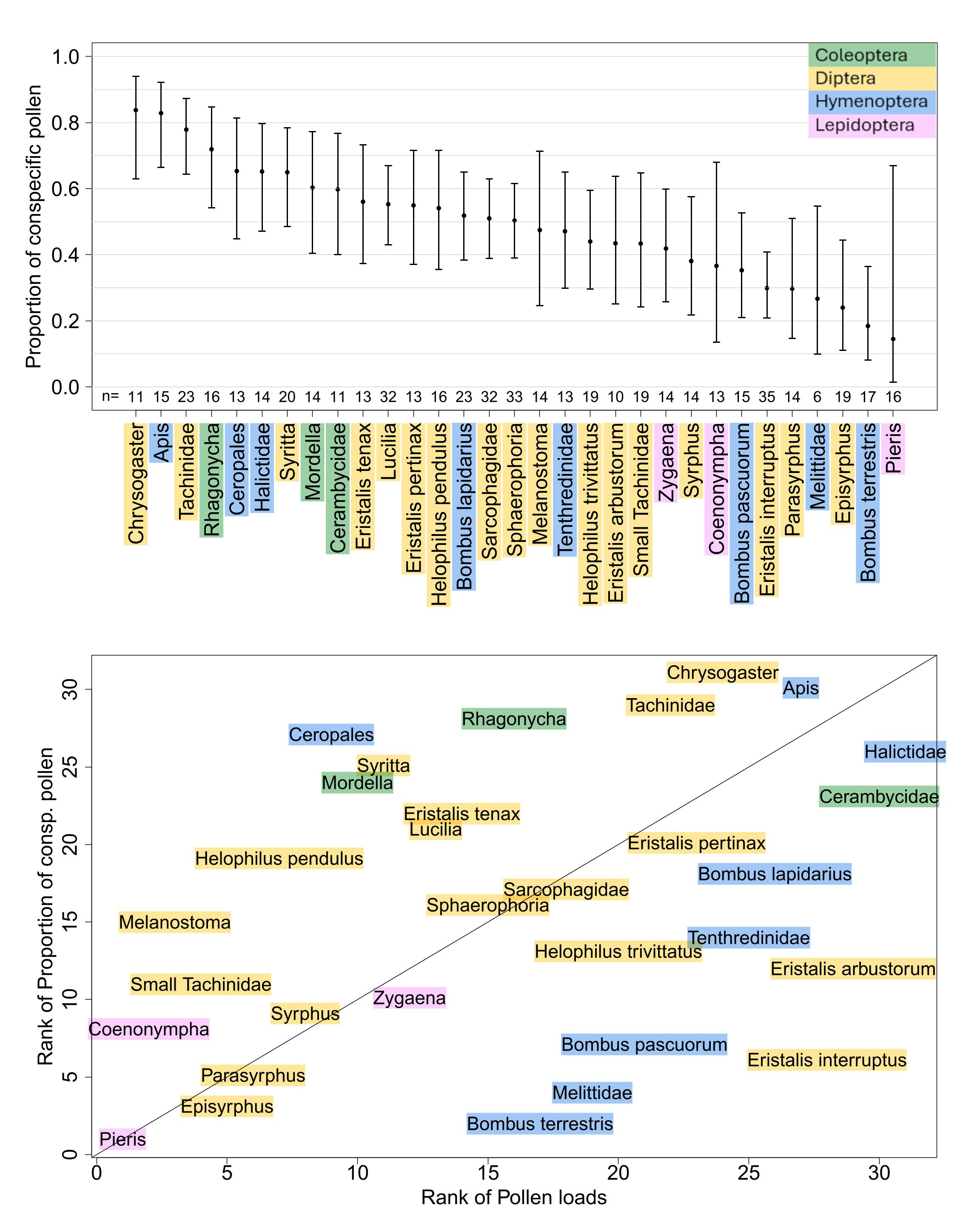

Supplement: plaf009_suppl_Supplementary_Materials_1_Tables_S1_Figures_S1-S2 [file plaf009_suppl_supplementary_materials_1_tables_s1_figures_s1-s2.zip › aobplants-24089S2R1-f04-z-4c.jpg]

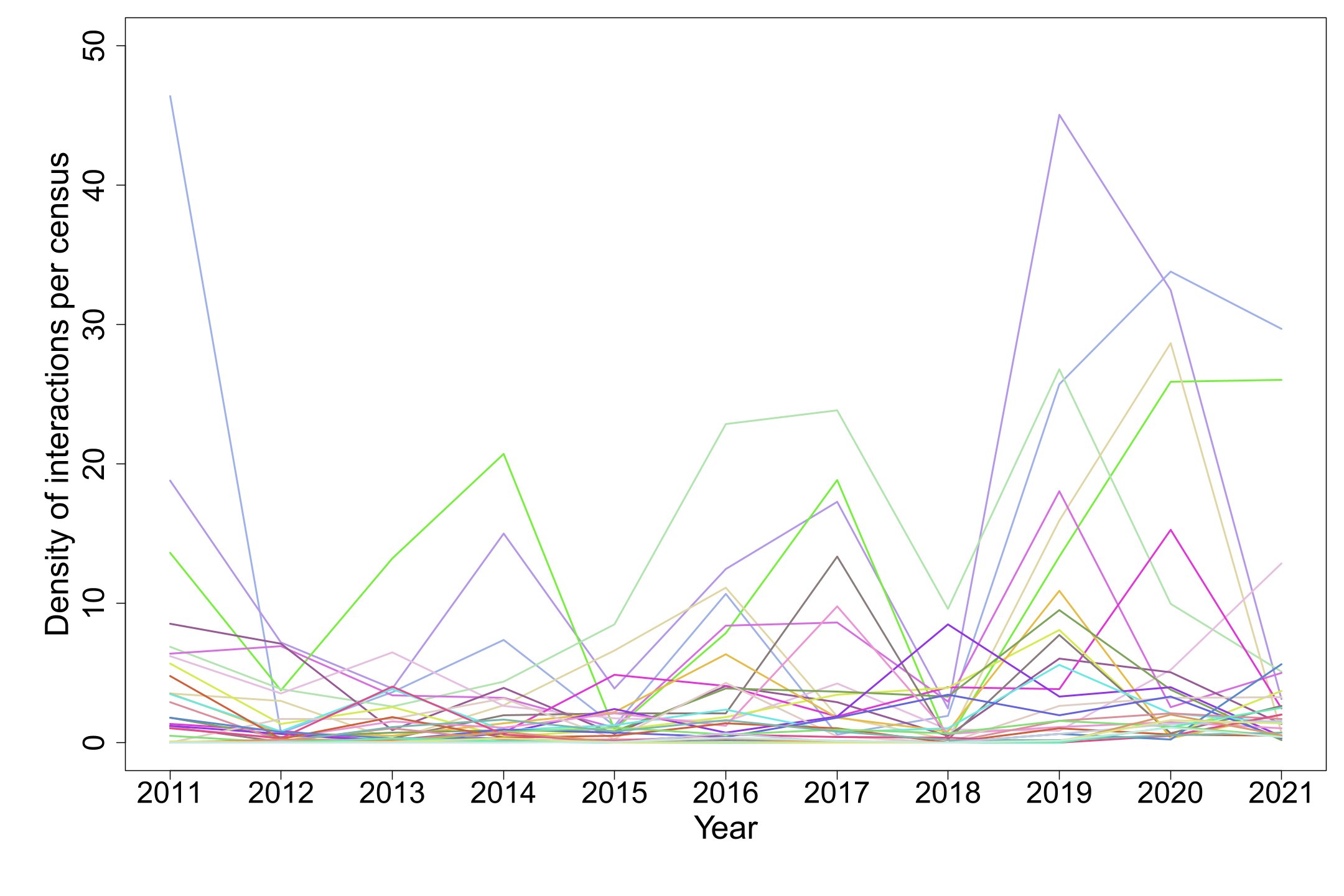

Supplement: plaf009_suppl_Supplementary_Materials_1_Tables_S1_Figures_S1-S2 [file plaf009_suppl_supplementary_materials_1_tables_s1_figures_s1-s2.zip › aobplants-24089S2R1-f05-z-4c.jpg]

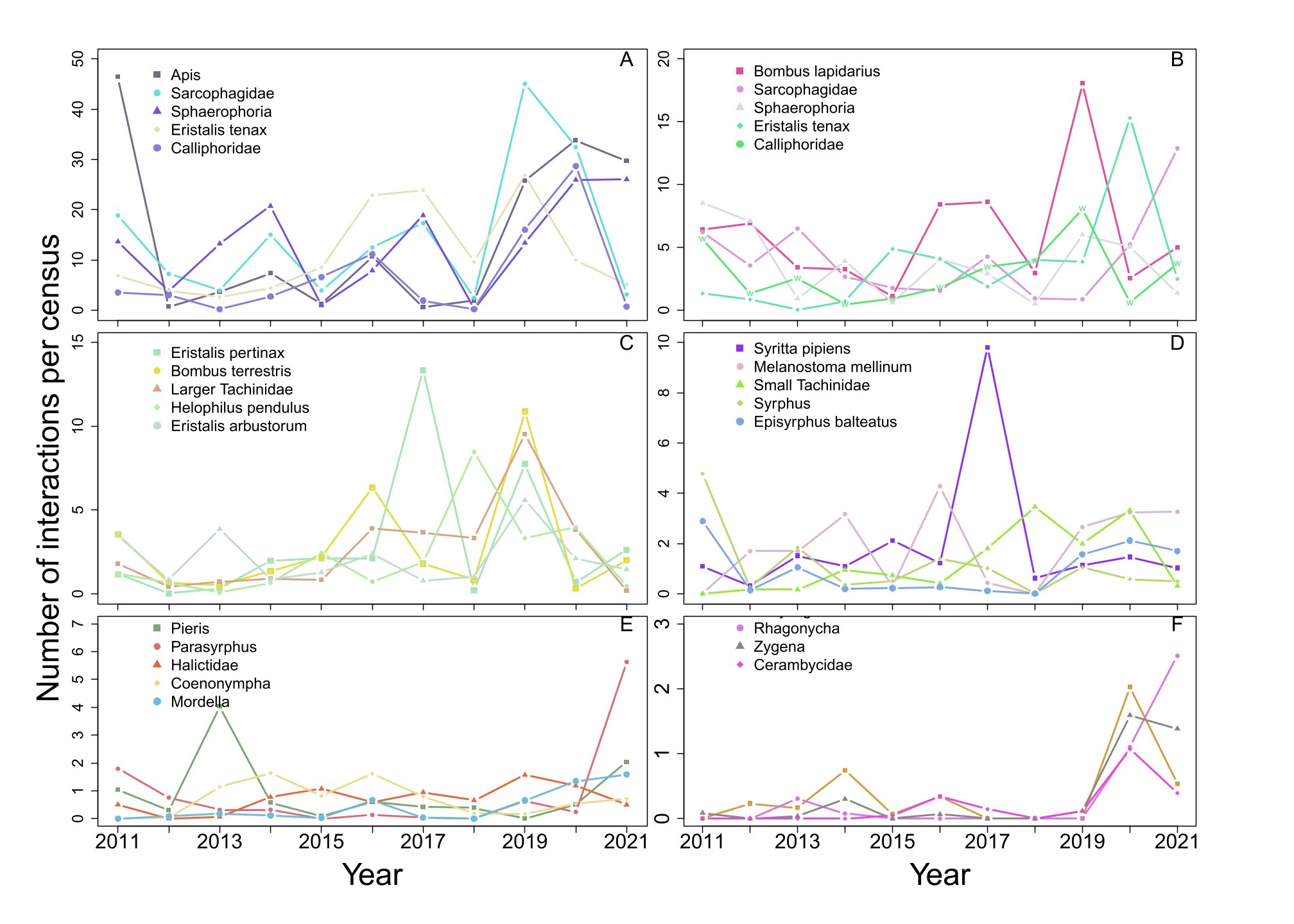

Supplement: plaf009_suppl_Supplementary_Materials_1_Tables_S1_Figures_S1-S2 [file plaf009_suppl_supplementary_materials_1_tables_s1_figures_s1-s2.zip › aobplants-24089S2R1-f06-z-4c.jpg]
